# HapX, an Indispensable bZIP Transcription Factor for Iron Acquisition, Regulates Infection Initiation by Orchestrating Conidial Oleic Acid Homeostasis and Cytomembrane Functionality in Mycopathogen Beauveria bassiana

**DOI:** 10.1128/mSystems.00695-20

**Published:** 2020-10-13

**Authors:** Yue-Jin Peng, Jia-Jia Wang, Hai-Yan Lin, Jin-Li Ding, Ming-Guang Feng, Sheng-Hua Ying

**Affiliations:** a Institute of Microbiology, College of Life Sciences, Zhejiang University, Hangzhou, China; Cornell University

**Keywords:** *HapX* gene, conidial reserve, phospholipid homeostasis, membrane functionality, virulence, *Beauveria bassiana*

## Abstract

In pathogenic filamentous fungi, conidial germination not only is fundamental for propagation in the environment but is also a critical step of infection. In the insect mycopathogen Beauveria bassiana, we genetically characterized the role of the basic leucine zipper (bZIP) transcription factor HapX (*BbHapX*) in conidial nutrient reserves and pathogen-host interaction. Ablation of *BbHapX* resulted in an almost complete loss of virulence in the topical inoculation and intrahemocoel injection assays. Comparative transcriptomic analysis revealed that *BbHapX* is required for fatty acid (FA)/lipid metabolism, and biochemical analyses indicated that *BbHapX* loss caused a significant reduction in conidial FA contents. Exogenous oleic acid could partially or completely restore the impaired phenotypes of the Δ*BbHapX* mutant, including germination rate, membrane integrity, vegetative growth, and virulence. *BbHapX* mediates fungal iron acquisition which is not required for desaturation of stearic acid. Additionally, inactivation of the Δ9-fatty acid desaturase gene (*BbOle1*) generated defects similar to those of the Δ*BbHapX* mutant; oleic acid also had significant restorative effects on the defective phenotypes of the Δ*BbOle1* mutant. A gel retarding assay revealed that BbHapX directly regulated the expression of *BbOle1*. Lipidomic analyses indicated that both *BbHapX* and *BbOle1* contributed to the homeostasis of phospholipids with nonpolar tails derived from oleic acid; therefore, exogenous phospholipids could significantly restore membrane integrity. These data reveal that the HapX-Ole1 pathway contributes to conidial fatty acid/lipid reserves and that there are important links between the lipid biology and membrane functionality involved in the early stages of infection caused by *B.* bassiana.

**IMPORTANCE** Conidial maturation and germination are highly coupled physiological processes in filamentous fungi that are critical for the pathogenicity of mycopathogens. Compared to the mechanisms involved in conidial germination, those of conidial reserves during maturation are less understood. The insect-pathogenic fungus Beauveria bassiana, as a representative species of filamentous fungi, is important for applied and fundamental research. In addition to its conserved roles in fungal adaptation to iron status, the bZIP transcription factor HapX acts as a master regulator involved in conidial virulence and regulates fatty acid/lipid metabolism. Further investigation revealed that the Δ9-fatty acid desaturase gene (*Ole1*) is a direct downstream target of HapX. This study reveals the HapX-Ole1 pathway involved in the fatty acid/lipid accumulation associated with conidial maturation and provides new insights into the startup mechanism of infection caused by spores from pathogenic fungi.

## Abstract (precis)

Conidial maturation and germination are highly coupled physiological processes in filamentous fungi that are critical for the pathogenicity of mycopathogens. Compared to the mechanisms involved in conidial germination, those of conidial reserves during maturation are less understood. The insect-pathogenic fungus Beauveria bassiana, as a representative species of filamentous fungi, is important for applied and fundamental research. In addition to its conserved roles in fungal adaptation to iron status, the bZIP transcription factor HapX acts as a master regulator involved in conidial virulence and regulates fatty acid/lipid metabolism. Further investigation revealed that the Δ9-fatty acid desaturase gene (*Ole1*) is a direct downstream target of HapX. This study reveals the HapX-Ole1 pathway involved in the fatty acid/lipid accumulation associated with conidial maturation and provides new insights into the startup mechanism of infection caused by spores from pathogenic fungi.

## INTRODUCTION

Unlike unicellular yeasts, filamentous fungi develop through an asexual sporulation process (1, 2), which produces numerous conidia and promotes fungal dispersal, survival, and evolution in ecosystems (3–5). Conidial germination is critical for the propagation of filamentous fungi and is indispensable for host infection by pathogenic fungi (6). Conidia are dormant cells whose cytoplasm is enclosed by the plasma membrane and cell wall (7). Two dormant forms (i.e., endogenous and exogenous dormancy) exist in fungal spores; the exit from exogenous dormancy to germination is triggered by water and involves the mobilization of endogenous nutrients (8). For instance, in Aspergillus niger, conidial germination occurs in the presence of water and requires additional sugars (e.g., glucose and mannose) for the formation of germ tubes (9). Conidia form and mature on the conidiophore, which is a process accompanied by the storage of nutrients (e.g., fatty acids [FAs] and lipids) (10). Fatty acids (FAs) act as the synthetic precursors of storage lipids and membranes (11). In addition to bound forms, various types of free FAs also accumulate in conidia (12, 13). The major pathways for FA synthesis are conserved among fungal species, including yeasts and molds (11), but the transcriptional regulation mechanisms underlying biosynthesis are divergent. For example, the OLE pathway, which involves the Δ9-fatty acid desaturase gene (*Ole1*), is critical for the synthesis of unsaturated fatty acids (UFAs). In Saccharomyces cerevisiae, the *Ole1* gene is controlled by the transcription factor Mga2, which contains an IPT/TIG domain (14). In Aspergillus flavus, the Zn_2_-Cys_6_ transcription factor FarA is required for activation of the *Ole1* ortholog (15). Filamentous fungi occupy a wide range of ecological niches (1). Thus, there is high diversity expected in transcription regulatory mechanisms of FA storage, particularly in conidia.

Filamentous entomopathogenic fungi (e.g., Beauveria bassiana and Metarhizium robertsii) are important biotic factors that regulate the arthropod populations of ecosystems and have enormous potential for the biological control of insect pests (16). Aerial conidia are the major dispersive and infectious propagules, both in nature and as the active ingredient in practical applications (17). Infection is initiated by conidial attachment to the host cuticle, followed by conidial germination (18). The infective hyphae breach the host cuticle and enter the hemocoel, where they develop into *in vivo* hyphal bodies through dimorphic transition (19). When the host is killed, the growing hyphae cover the cadaver and produce thick conidia responsible for subsequent infection cycles (20, 21). The conidia of entomopathogenic fungi germinate on oligotrophic insect cuticles, which represent special niches of host-fungus interaction. *B*. bassiana is a well-investigated filamentous entomopathogenic fungus, and its conidia perform exogenous dormancy and germinate after contact with water by mobilizing its endogenous nutrients (22). B. bassiana accumulates many FAs in its conidia (23). Therefore, *B.* bassiana could be an ideal representative species of filamentous fungi for exploring the mechanisms involved in FA accumulation in the conidia of mycopathogens.

In cells, gene transcription is regulated by various transcription factors (TFs). The basic leucine zipper (bZIP) family of TFs is present in all eukaryotic organisms and involved in extensive cell physiologies. The bZIP domain consists of the basic and leucine zipper regions (24). Among diverse bZIP-type TFs, HapX is characterized by a CCAAT binding complex (CBC)-interacting domain at the N terminus (25). The CBC is a highly conserved transcriptional initiation regulator in eukaryotes. In the filamentous fungus Aspergillus nidulans, the core structure of the CBC consists of three subunits: HapB, HapC, and HapE (or in the case of S. cerevisiae, Hap2/3/5, respectively) (26). The A. nidulans CBC recruits HapX, which shares a domain with the CBC-recruited Hap4 in S. cerevisiae (25). In S. cerevisiae, Hap4 is essential for transcriptional activation of respiration (27). Based on the investigations of *Aspergillus* species, the CBC contributes to diverse processes (e.g., metabolism and development) in filamentous fungi (28). The roles of HapX and action mode have been best characterized in Aspergillus fumigatus, a human mycopathogen. HapX mediates iron homeostasis, which is essential for fungal physiology. When iron abundance is in excess, HapX activates vacuolar iron storage to enhance fungal resistance to the high-iron conditions (29). During iron limitation or starvation, HapX represses the iron-consuming pathways and enhances the iron-uptake pathways by activating siderophore synthesis. *HapX* loss attenuates the virulence of A. fumigatus, which indicates that HapX is required for fungal adaption to iron starvation in host niches (30). Additionally, transcription factor HapX has been investigated in other fungal species: A. nidulans (25), Aspergillus terreus (31), Arthroderma benhamiae (32), Fusarium oxysporum (33), Verticillium dahliae (34), Candida albicans (35), and Cryptococcus neoformans (36). In all these species, *HapX* is required for adaptation to iron starvation and in most also for iron resistance (except for C. albicans). In A. fumigatus and C. albicans, direct repression of genes involved in iron-consuming pathways during iron starvation has been revealed via chromatin immunoprecipitation (29, 35). However, robust data regarding the roles of HapX in entomopathogenic fungi are still not available.

In this study, a B. bassiana bZIP-type TF gene (BBA_06576) was recognized as gene *HapX* (*BbHapX*) and functionally analyzed. The *HapX* role in fungal adaptation to iron availability is conserved for *B.* bassiana. Importantly, our data revealed that *BbHapX* regulates conidial UFA storage and membrane functionality required for fungi to successfully infect insect hosts.

## RESULTS

### *BbHapX* is crucial for fungal virulence.

A BLASTP search using the amino acid sequence of A. nidulans HapX (AnHapX) (25) revealed a single significant match (1e−071), BBA_06576, in *B.* bassiana (37). *B.* bassiana *HapX* (*BbHapX*) is encoded by an open reading frame (ORF) of 1,950 bp with a deduced protein of 649 amino acids. BbHapX contains two typical traits in this class of transcription factors, including a CBC-interacting domain and a bZIP domain (see Fig. S1A and B in the supplemental material). Also, BbHapX contains a cysteine-rich-region containing an iron-sensing motif (CGFCN5CNC) which is prevalent in the HapX proteins from filamentous fungi (29) (Fig. S1C). Sequence similarity between two CBC-binding proteins was rather low, and BbHapX displays only 7.40 to 46.8% overall identity to the orthologs from other fungi (Fig. S1D).

10.1128/mSystems.00695-20.1 FIG S1 Sequence identity matrix for HapX transcription factors in fungi. (A) A diagram showing the domain architecture in the BbHapX sequence. CBC, CCAAT-binding complex. bZip domain, basic-leucine zipper domain. (B and C) The characterized HapX sequences from fungi were aligned, and the identical residues among the CBC-binding domains (B) are indicated by asterisks, and the motif CGFCN5CNC is framed (C). (D) The similarity between any two sequences was analyzed with the software of BioEdit (version 7.0.9.0). The colors from yellow to green indicate the different HapX similarities between two sequences. The HapX accession numbers are from the following fungi: *Arthroderma benhamiae* (Ab), D4AQY2; Aspergillus fumigatus (Af), Q4WER3; A. nidulans (An), AB052971; Beauveria bassiana (Bb), EJP64582; Fusarium oxysporum (Fo), KNB05236; Verticillium dahliae (Vd), EGY23584; Cryptococcus neoformans (Cn), AFR94904; Kluyveromyces lactis (Kl), AAD20134; and Saccharomyces cerevisiae (Sc), P14064. Download FIG S1, TIF file, 1.1 MB. Copyright © 2020 Peng et al. 2020 Peng et al. This content is distributed under the terms of the Creative Commons Attribution 4.0 International license.

To elucidate the roles of *BbHapX* in B. bassiana, a target deletion mutant of *BbHapX* was generated by homologous recombination, and the effect of gene loss was rescued by ectopic integration of the entire *BbHapX* gene into the *ΔBbHapX* strain (Fig. S2).

10.1128/mSystems.00695-20.2 FIG S2 Molecular manipulation for functional analysis of gene in Beauveria bassiana. (A) Diagram indicating the strategy of homologous recombination for gene disruption in fungal genome. “X” means two genes to be investigated, i.e., *BbHapX* and *BbOle1*. (B to E) The gene disruption and reconstructed mutants were screened by PCR (B and D), and were further confirmed by Southern blot analysis (C and E). In Southern blotting, restriction enzymes (En) for DNA digestion were SacI/NcoI and BamHI/HindIII, respectively. Download FIG S2, TIF file, 0.3 MB. Copyright © 2020 Peng et al. 2020 Peng et al. This content is distributed under the terms of the Creative Commons Attribution 4.0 International license.

**FIG 1:**
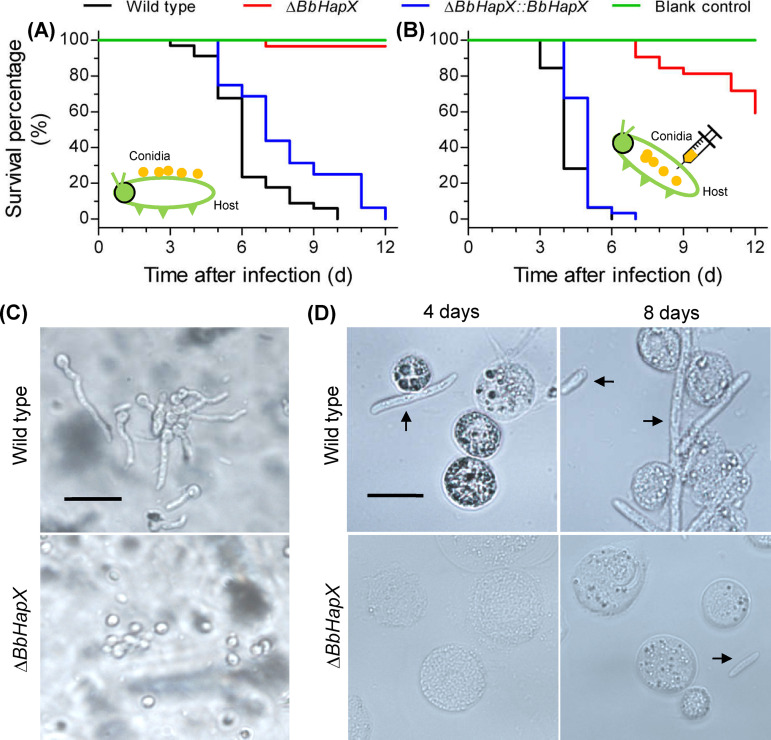
Effects of the *BbHapX* mutation on the virulence of Beauveria bassiana against Galleria mellonella. (A) Survival curve for insect hosts after topical inoculation with conidial suspension (10^7^ cells/ml) of the WT, Δ*BbHapX*, and complemented strains. (B) Survival curve for insect hosts after injection with 5 μl of conidial suspension (10^6^ cells/ml) of the three strains mentioned in panel A. Statistical analysis of survival data was performed by a Kaplan-Meier pairwise comparison using a log-rank test. (C) Conidial germination on the locust hindwing, which was used to mimic the insect cuticle. Conidia of the WT germinated well, while the mutant had barely germinated at 1 day postincubation. (D) Representative images of hyphal bodies in hemolymph. The rod-like cells are hyphal bodies (indicated with black arrows), and the round cells are host hemocytes.

Ablation of *BbHapX* severely attenuated fungal pathogenicity. In the cuticle-invasion route (Fig. 1A), the insects infected with the Δ*BbHapX* strain exhibited very low mortality (<5%) at 12 days postinfection (DPI), whereas both wild-type (WT) and complementation strains killed all insects. The survival curve of the Δ*BbHapX* strain was extremely significant compared to those of the WT and complementation strains (*P < *0.0001). The median lethal time (LT_50_) values for the WT and complementation strains were 6 and 7 days, respectively. When bypassing the host cuticle, Δ*BbHapX* mutant displayed 31% mortality at 12 DPI, which was highly significantly different from those of the WT and complementation strains (100%) (*P < *0.0001) (Fig. 1B). The LT_50_ values for the latter two strains were 4 and 5 days, respectively. Additionally, conidia of the WT strain germinated well and developed into germ tubes on the host cuticles; however, most conidia of the Δ*BbHapX* mutant did not germinate (Fig. 1C). In host hemocoel, conidia of the WT strain developed into hyphal bodies 4 DPI, whereas the Δ*BbHapX* mutant formed hyphal bodies at 8 DPI (Fig. 1D). These results demonstrated that *BbHapX* is critical for fungal growth in the host hemocoel and, in particular, for the fungal ability to penetrate the host exoskeleton.

### *BbHapX* activates the genes involved in lipid/FA metabolism.

*BbHapX* deficiency resulted in 1,143 differentially expressed genes (DEGs), with 421 upregulated (∼4.1% of the genome) and 722 downregulated (∼7.0% of the genome) genes in B. bassiana. Additionally, 10 genes were completely repressed in the Δ*BbHapX* mutant (Data Set S1).

10.1128/mSystems.00695-20.5 DATA SET S1 The differentially expressed genes (DEGs) in comparison of WT/Δ*BbHapX* strains during conidiation process. Download Data Set S1, XLSX file, 0.1 MB. Copyright © 2020 Peng et al. 2020 Peng et al. This content is distributed under the terms of the Creative Commons Attribution 4.0 International license.

**FIG 2:**
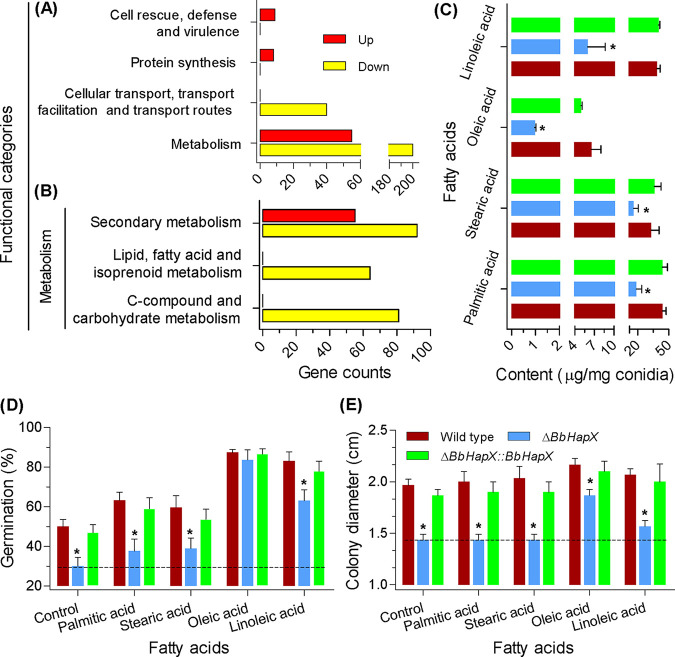
Ablation of *BbHapX* impairs fatty acid metabolism in B. bassiana. (A) Functional distribution analysis of the *BbHapX*-mediated transcriptome. Differentially expressed genes (DEGs) were uncovered by comparative transcriptomics between the WT and gene disruption mutant strains. Enrichment analysis indicated that these DEGs were overrepresented in six functional categories. (B) The secondary categories within the main category of “metabolism.” Categories involved in fatty acid and carbohydrate metabolism were enriched in the downregulated DEGs. Yellow and red bars indicate the down- and upregulated genes, respectively. (C) Fatty acid content in conidia. Four free fatty acids (stearic, palmitic, oleic, and linoleic) dominated in conidia. Levels of the four fatty acids declined in the Δ*BbHapX* mutant strain. (D) Effects of exogenous fatty acids on conidial germination. Conidia of the indicated strain were inoculated on water agarose (WA) plates containing various fatty acids. After 1 day of incubation at 25°C, the germination percentage was determined. The WA plates were used as a control. (E) Effects of exogenous fatty acids on fungal vegetative growth. On the basis of SDAY medium (control), various fatty acids were included in the plates. A conidial suspension (1 μl, 10^6^/ml) was inoculated on the plates and incubated at 25°C. Seven days later, the colony diameter was examined. The dotted line indicates the phenotypic values of the Δ*BbHapX* mutants. Asterisks indicate a significant difference between the Δ*BbHapX* mutant and WT or complementation strain (Tukey’s honestly significant difference [HSD]: *P *< 0.05). Error bars: standard deviation.

At the main level of functional categories (Fig. 2A), the upregulated DEGs were significantly overrepresented in the terms of metabolism, protein synthesis, and cell rescue (Data Set S2), whereas the downregulated DEGs were enriched in the categories of metabolism and cellular transport (Data Set S3). In the overlapped subcategory of metabolism (Fig. 2B), the upregulated DEGs were enriched only in the term of secondary metabolism, while the downregulated DEGs had additional terms of lipid/fatty acid/isoprenoid metabolism and carbohydrate metabolism. In budding yeast, *Ole1* encodes a Δ9-fatty acid desaturase which is indispensable for the biosynthesis of UFA (14). In B. bassiana, there are two orthologs of *Ole1* (i.e., BBA_07664 and BBA_09419) whose expression was significantly repressed in the *ΔBbHapX* mutant (Data Set S1).

10.1128/mSystems.00695-20.6 DATA SET S2 Enrichment analysis of the upregulated DEGs associated with conidiation. Download Data Set S2, XLSX file, 0.01 MB. Copyright © 2020 Peng et al. 2020 Peng et al. This content is distributed under the terms of the Creative Commons Attribution 4.0 International license.

10.1128/mSystems.00695-20.7 DATA SET S3 Enrichment analysis of the downregulated DEGs associated with conidiation. Download Data Set S3, XLSX file, 0.01 MB. Copyright © 2020 Peng et al. 2020 Peng et al. This content is distributed under the terms of the Creative Commons Attribution 4.0 International license.

### *BbHapX* is required for the metabolic homeostasis of FAs.

Ablation of *BbHapX* resulted in a significant reduction in the content of four FAs (Fig. 2C). The reductions for stearic and palmitic acids were 51% and 57%, respectively, whereas the reductions for oleic and linoleic acids were 87% and 84%, respectively. Exogenous UFAs could partially restore conidial germination on water agar (WA) plates (Fig. 2D) and vegetative growth on nutrient plates (Fig. 2E); however, the saturated fatty acids had no significant effects. In addition, glucose had no restoration effects on the germination percentage of the *BbHapX* mutant strain grown on the WA plates. This suggests that the *BbHapX*-mediated FA metabolism is not involved in energy supply during conidial germination.

### Exogenous OA recovers membrane integrity and virulence.

**FIG 3:**
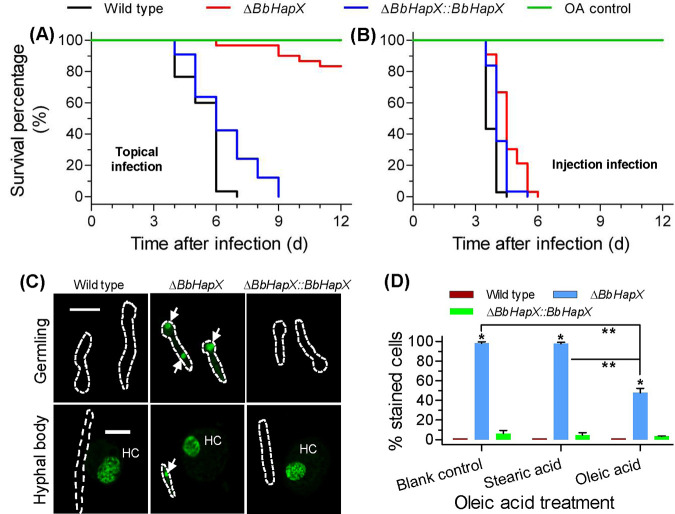
Recovery effects of exogenous oleic acid (OA). A feeding test was performed on the WT, Δ*BbHapX* mutant, and complemented strains. (A and B) OA feeding had significant effects on fungal virulence in the cuticle infection (A) and intrahemocoel injection (B) tests. Conidia were cultured on SDAY plates and suspended in 0.02% Tween 80 solution containing 0.2% OA (final concentration). OA was added to conidial suspension by vigorous vortexing. All methods for the bioassay and statistical analysis were the same as those in Fig. 1. Tween solution-OA was used as the control. (C) SYTOX Green staining of B. bassiana germlings and hyphal bodies. The stained nuclei (green) in the fungal cells are indicated with arrows. The dotted lines indicate the outlines of fungal cells. HC indicates the hemocyte in the host hemolymph. Bar, 10 μm. (D) Effect of OA treatment on the percentage of SYTOX-stained cells among germlings. Almost all germlings of the Δ*BbHapX* mutant strain were stained by SYTOX Green, while almost all WT strain cells could not be stained. Exogenous OA significantly reduced the percentage of stained cells (PSC) in the *BbHapX*-null strain. Stearic acid was used as a fatty acid control. Asterisks indicate a significant difference between the gene disruption mutant and WT or complementation strain. Double asterisks indicate a significant difference in the PSC of Δ*BbHapX* mutant between the OA treatment and control (Tukey’s HSD: *P* < 0.05). Error bars: standard deviation.

When topically inoculating conidia, oleic acid (OA) significantly enhanced the virulence of the Δ*BbHapX* mutant, with approximately 20% mortality at 12 DPI (Fig. 3A). Its survival curve was extremely distinct from those of the WT and complementation strains (*P < *0.0001). The LT_50_ value for the latter two strains was 6 days. Strikingly, when adding oleic acid in the intrahemocoel injection bioassay, the Δ*BbHapX* mutant killed all insect hosts at 6 DPI, whereas the WT strain displayed 100% mortality at 4 DPI (Fig. 3B). The LT_50_ values for the WT and mutant strains were 3.5 and 4.5, respectively. These results indicated that exogenous oleic acid decreased the difference in mortality and LT_50_ value between the WT and Δ*BbHapX* mutant strains.

Loss of *BbHapX* led to poor membrane integrity in germlings and *in vivo* blastospores (Fig. 3C). Without oleic acids, nearly all germlings of *ΔBbHapX* mutant were stained by SYTOX, while only 1% of the WT cells were permeable to this dye. However, exogenous oleic acids significantly reduced the percentage of the stained cells (PSC) (∼50%) (Fig. 3D). Stearic acid could not significantly reduce the PSC value for the Δ*BbHapX* mutant. These data show that disruption of *BbHapX* results in impaired membrane integrity, which could be restored by supplying oleic acids.

### *BbHapX* contributes to fungal adaptation to iron starvation.

**FIG 4:**
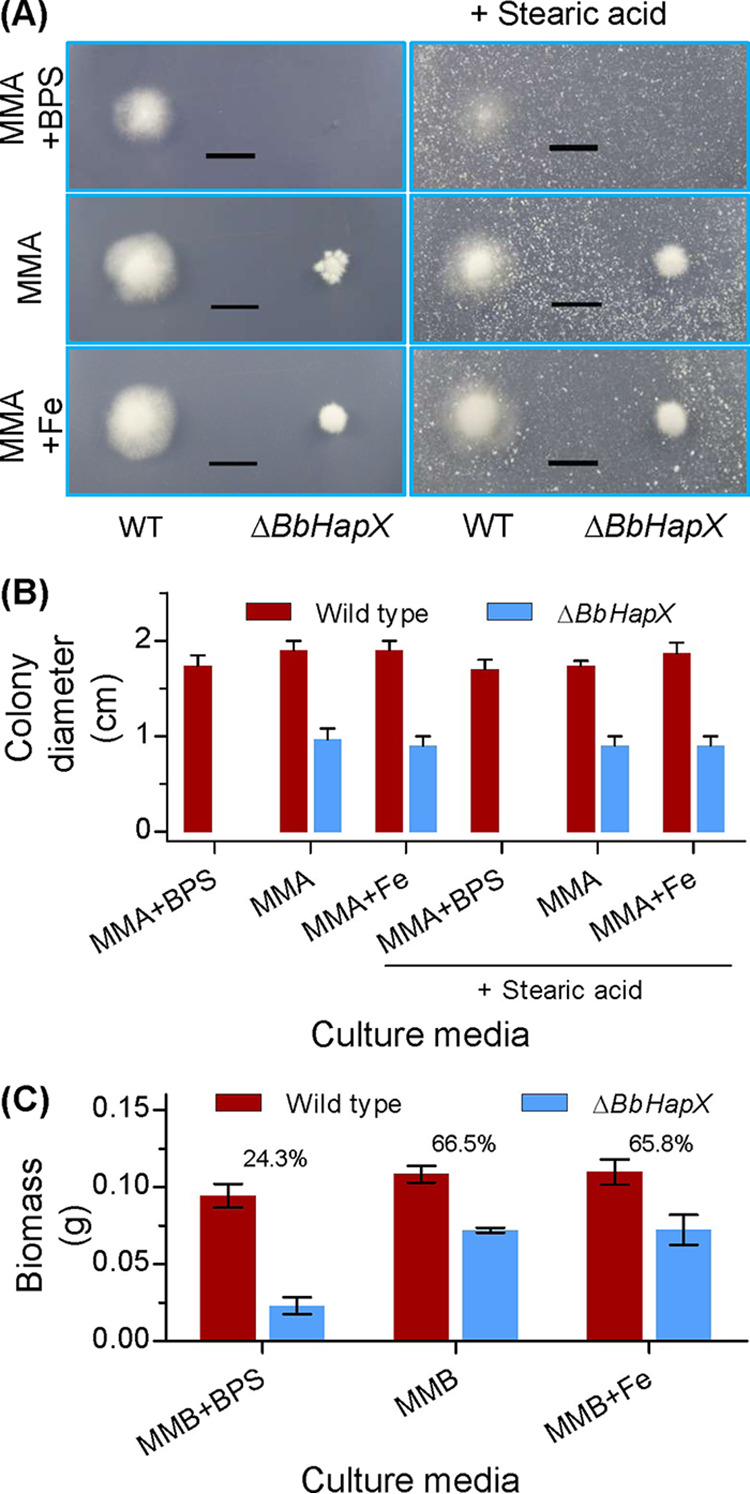
Effects of iron availability on fungal growth. (A) Aerial growth on the defined media. Minimal nutrient agar (MMA) plates were included with bathophenanthroline disulfonate (BPS) (0.2 mM) or 30 μM ferrous iron (Fe). Meanwhile, stearic acid was supplemented in plates at the final concentration of 0.3%. Conidial suspension was inoculated on the plates and cultured at 25°C. (B) Seven days later, the colony diameter was examined. (C) Submerged growth in the defined broth. The agarose in MMA was omitted, generating MMB broth. The chemicals were included as mentioned above. After inoculation, the broth was cultured at 25°C with shaking. Six days later, mycelia were harvested and dried. The number on the column is the biomass ratio of WT to Δ*BbHapX* mutant. Scale bars in panel A represent 1 cm.

On the minimal nutrient agar (MMA) plates (Fig. 4A and B), The Δ*BbHapX* mutant displayed a colony which was significantly smaller than that of the WT strain. This difference was not changed by supplementing sufficient iron (MMA+Fe). Notably, in the presence of the iron chelator bathophenanthroline disulfonate (BPS), the WT strain was not significantly influenced, but the Δ*BbHapX* mutant did not form a visible colony. All these defects could not be recovered by exogenous stearic acid (Fig. 4A). Under submerged conditions (Fig. 4C), the relative levels of biomass (RLB) between the WT and mutant strains were similar between MMB and MMB+Fe broth. The RLB value was dramatically reduced in the presence of BPS. Additionally, on the WA plates containing 30 μM ferrous iron, the germination percentages for the WT and Δ*BbHapX* mutant strains were 51.7% ± 3.5% (mean ± standard deviation) and 30.4% ± 3.2%, respectively. The difference between two strains was similar to that on WA plates. On the WA plates including stearic acid and BPS, the germination percentages for the WT and Δ*BbHapX* mutant strains were 58.7% ± 4.6% and 38.7%, respectively. The difference between two strains was not distinct from that on the WA plates with stearic acid. These results indicated that ambient iron availability had no significant effects on conidial germination and radial growth of the Δ*BbHapX* mutant.

### *BbOle1* is a direct target of transcription factor BbHapX.

**FIG 5:**
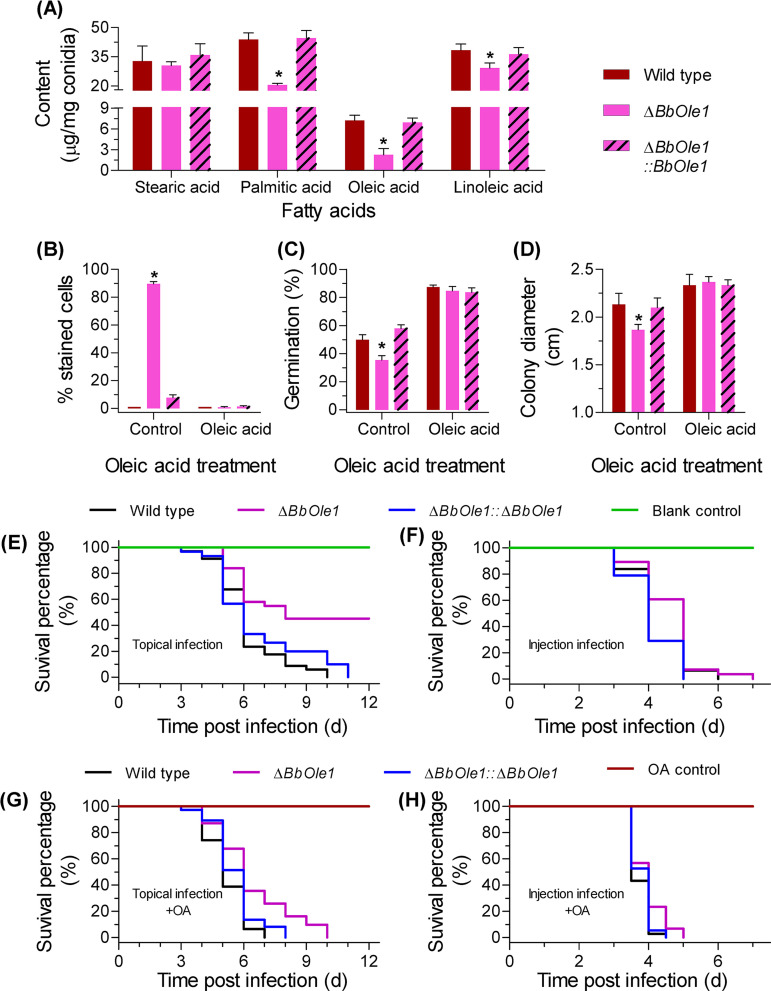
Functional analyses of *BbOle1* in B. bassiana. (A) Fatty acid content in conidia. Gene loss resulted in a significant reduction in palmitic, oleic, or linoleic acid content. (B) Percentage of SYTOX-stained germlings. (C) Conidial germination on the water agarose plates. Conidial suspension was smeared on the plates and incubated at 25°C for 1 day. (D) Vegetative growth on SDAY plates. Conidial suspension was dotted on the plates, and the colony diameter was examined at 7 days postincubation at 25°C. (E to H) Conidial virulence was evaluated with topical infection (E and G) and intrahemocoel injection (F and H) bioassays. Survival data were plotted as Kaplan-Meier results, and statistical analyses were the same as those in Fig. 1. Exogenous oleic acid (OA) significantly reduced the phenotypic difference between the WT and Δ*BbHapX* mutant strains observed in panels B to H. Asterisks on columns denote a significant difference between the Δ*BbOle1* and WT or complementation strains (Tukey’s HSD: *P* < 0.05). Error bars: standard deviation.

Only the gene of BBA_07664 (named *BbOle1*) was successfully disrupted. Biochemical analysis indicated that *BbOle1* was required for the homeostasis of FA (Fig. 5A). Disruption of *BbOle1* resulted in a slight decrease in linoleic acid content (24%) and a modest reduction in the content of palmitic acids (58%) and oleic acids (63%). However, *BbOle1* loss had no significant effect on conidial storage of stearic acids. Disruption of *BbOle1* resulted in 90% of the cells being stained by SYTOX. After oleic acid feeding, there was no significant difference in the PSC values among the WT, gene disruption mutant, and complementation strains (Fig. 5B). This result indicated that *BbOle1* contributes to membrane integrity, which could be restored by additional oleic acids. A significantly lower germination of the Δ*BbOle1* mutant conidia (35.3% ± 3.2%) was observed on the WA plates compared to the WT parent (50.0% ± 3.6%) (Fig. 5C). On Sabouraud dextrose agar (SDAY) plates, the disruption mutant displayed a slight growth defect. Nevertheless, oleic acid feeding completely eliminated the growth difference between the WT and gene disruption mutant strains (Fig. 5D). *BbOle1* ablation significantly weakened conidial virulence in two bioassays. In the topical bioassay (Fig. 5E), survival curves for the WT and Δ*BbOle1* mutant strains were extremely significant (*P < *0.0001), and the LT_50_ values for these two strains were 6 and 8 days, respectively. Though oleic acids did not decrease the LT_50_ for the Δ*BbOle1* mutant, the mutant strain killed all insects at 12 DPI (Fig. 5G). The LT_50_ values for the WT and Δ*BbOle1* mutant strains were 6 days. In the intrahemocoel injection bioassays (Fig. 5F), the LT_50_ values for the WT and Δ*BbOle1* mutant strains were 4 and 5 days, respectively. Adding oleic acids resulted in the reduced difference in LT_50_ between the WT (3.5 days) and Δ*BbOle1* mutant (4 days) (Fig. 5H). Apparently, lack of *BbOle1* results in significantly lower attenuation compared to lack of *BbHapX*, which indicates that the role of *BbHapX* in virulence of B. bassiana is not limited to regulation of *BbOle1*.

**FIG 6:**
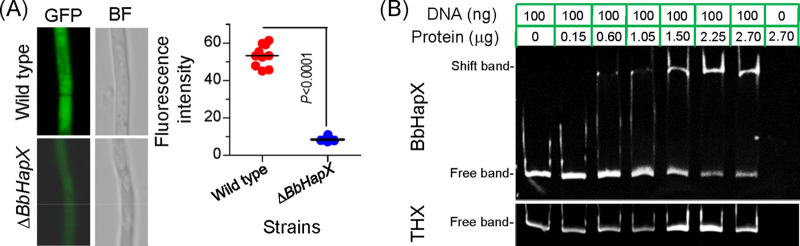
*BbHapX* contributes to transcription of *BbOle1*. (A) Transcription activity of the *BbOle1* promoter. The green fluorescent protein (GFP) gene was under the control of the *BbOle1* promoter. The hybrid DNA fragment was transformed into the WT and Δ*BbHapX* mutant strains. The green signals and mycelial morphology were recorded under the fluorescent and bright field (BF), respectively. There is a significant difference in fluorescence intensity between the WT and Δ*BbHapX* mutant strains (*t* test). (B) Gel shift assay for the binding activity of BbHapX with the promoter of *BbOle1*. The soluble BbHapX was prepared by the heterogenous expression of a hybrid gene, *BbHapX*::*Thioredoxin*. A 20-μl reaction system contained the promoter DNA (100 ng) and various proteins (0.3 to 2.7 μg). The DNA-protein complex was resolved in a 6% polyacrylamide gel. The shifted DNA bands were visualized by ethidium bromide stain. Thioredoxin (THX) protein was used as a control to examine whether the fusion protein tag bound to the promoter.

The fluorescence intensity in WT was significantly higher than that in the Δ*BbHapX* mutant (Fig. 6A). Furthermore, BbHapX was prepared in a heterologous expression system with a fused tag of thioredoxin (Fig. S3). During electromobility shift assay (EMSA), the fused protein bound to the promoter region of *BbOle1*, while thioredoxin had no binding activity to the promoter (Fig. 6B). This result indicated that the *BbOle1* gene functions as a direct target of the BbHapX factor.

10.1128/mSystems.00695-20.3 FIG S3 Heterologous expression and purification of BbHapX. The coding sequence of *BbHapX* was cloned into the pET32a expression vector (Novagen), and the fusion protein had two tags (i.e., His and Thx). The hybrid gene was expressed in the Rosetta strain (Novagen), and the target protein was purified by Ni-chelating column affinity chromatography. The purified protein was dialyzed, and its purity was confirmed by polyacrylamide gel electrophoresis. Lane 1, molecular marker. Lane 2, purified protein. Download FIG S3, TIF file, 0.03 MB. Copyright © 2020 Peng et al. 2020 Peng et al. This content is distributed under the terms of the Creative Commons Attribution 4.0 International license.

### *BbHapX* and *BbOle1* are required for phospholipid homeostasis.

**FIG 7:**
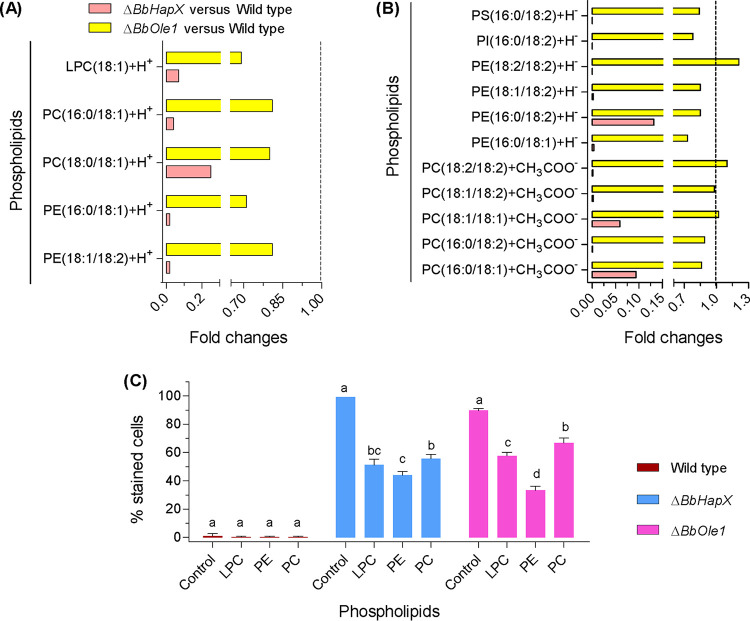
Comparative lipidomics between the wild-type (WT) and Δ*BbHapX* or Δ*BbOle1* mutant strains. (A and B) Phospholipids might exist in their cationic (A) or anionic (B) forms. The intensity of the indicated molecule was quantified by mass spectrum. The ratios of WT to mutant were calculated as relative levels. *BbHapX* has a more significant effect on conidial phospholipid profiles than *BbOle1*. (C) Effects of exogenous phospholipids on the percentage of SYTOX-stained germlings. Phospholipid feeding significantly reduced the percentage of stained cells in both the Δ*BbHapX* and Δ*BbOle1* mutant strains. Different lowercase letters on bars indicate significant differences in percentage of the indicated strain fed with different phospholipids. LPC, lysophosphatidylcholine; PC, phosphatidylcholine; PE, phosphatidylethanolamine; PI, phosphatidylinositol. Error bars: standard deviation.

Lipid molecules can exist in their cationic or anionic forms, and certain lipids exist in both forms (e.g., PC16:0/18:1) (Data Set S4). In the cationic profiles (Fig. 7A), the contents of five phospholipids with oleic acid (OA) as an FA chain (e.g., phosphatidylethanolamine [PE] and phosphatidylcholine [PC]) were notably reduced in the Δ*BbHapX* mutant by approximately 75 to 95% compared with WT. Meanwhile, the *BbOle1* loss resulted in a slight to modest reduction (approximately 20 to 30%) in all detected lipids. As for anionic forms (Fig. 7B), *BbHapX* mutation caused significant reductions in the contents of 11 phospholipids (e.g., PE and PC). In the Δ*BbOle1* mutant strain, seven phospholipids (e.g., PE18:1/18:2) had slight decreases and the other four lipids (e.g., PC18:1/18:2) had no significant changes. These results showed that *BbHapX* and *BbOle1* had overlapping roles in phospholipid generation, but *BbHapX* had a greater effect than *BbOle1*.

10.1128/mSystems.00695-20.8 DATA SET S4 The intensities of ionized phospholipids with unsaturated fatty acids as hydrophobic chains. Download Data Set S4, XLSX file, 0.01 MB. Copyright © 2020 Peng et al. 2020 Peng et al. This content is distributed under the terms of the Creative Commons Attribution 4.0 International license.

As shown in Fig. 7C, nearly all germlings of the WT strain were not stained by SYTOX, and exogenous phospholipids had no effect on the PSC values. Feeding tests indicated that exogenous phospholipids could reduce the PSC values in two gene disruption mutants. In the Δ*BbHapX* mutant, lysophosphatidylcholine (LPC) and PC resulted in an approximately 48% and 43% reduction in PSC values, respectively. As for the Δ*BbOle1* strain, these two chemicals caused an approximately 36% and 26% reduction in the PSC, respectively. PE had a greater effect in reducing the PSC values, with an approximately 56% and 63% reduction in the Δ*BbHapX* and Δ*BbOle1* mutant strains, respectively. These data indicated that exogenous phospholipids could reduce cytomembrane permeability to SYTOX, and the decreasing degrees of the PSC values were very similar between two gene disruption mutants fed with the indicated phospholipid.

## DISCUSSION

In filamentous fungi, transcription factor HapX plays a vital role in the fungus’ ability to handle iron starvation in the environment (38) by orchestrating complex metabolic pathways (e.g., siderophore synthesis) (39, 40). In B. bassiana, *BbHapX* is also indispensable for fungal survival under iron starvation. These facts reinforce that *HapX* is a conserved regulator for fungal iron acquisition, and the question as to whether the *HapX*-mediated pathways are present in B. bassiana is still open. Conidial germination is an indispensable process in the initial stage of fungal pathogen infection and is highly related to the conidial maturation process (6). Strikingly, besides its roles in fungal adaptive capacity to ambient iron status, B. bassiana *HapX* acts as an essential transcriptional regulator of infection initiation by regulating FA/phospholipid synthesis during conidial maturation.

In filamentous pathogenic fungi (e.g., Magnaporthe grisea), the reserve lipids are hydrolyzed into FAs and glycerol for conidial germination, which is critical for fungal virulence (41). However, the regulatory mechanisms for lipid/FA storage are still unknown for conidia. In this study, HapX, a bZIP transcription factor, is established as an essential regulator for FA homeostasis in conidia and fungal virulence. A previous study indicated that B. bassiana conidia contain stearic, palmitic, oleic, linoleic, and linolenic acids (23). Our B. bassiana strain accumulates only the first four FAs, which further suggests that accumulation of lipids/FAs varies among different cell types and fungal species (13). B. bassiana conidia can germinate upon contact with water in the absence of external nutrients (22), but external FAs can significantly enhance conidial germination. Conidia require nutrients for germination, and in some phytopathogenic fungi, germination initiates after the conidia adhere to the hydrophobic surface (42) and requires the chemicals exuded by the host plant (43). In B. bassiana, *HapX* is required for conidial germination on oligotrophic host cuticle, and its loss could be recovered by exogenous UFAs. These results suggest that germination initiation is rather complicated and varies among fungi. During interaction of the fungus and the host, *BbHapX* contributes to its almost complete virulence during topical infection and partial virulence in intrahemocoel infection. The surface of the insect cuticle is deficient in nutrients (22), whereas the host hemolymph is rich in nutrients, including FAs (e.g., oleic acid) (44). These facts readily explain why exogenous oleic acid restores partial fungal virulence during cuticle penetration and complete virulence via direct infection. Oleic acid could recover the full conidial germination rate but only partial virulence in the topical application bioassay. This might be due to the complexity of host cuticles (18) and the multiple roles of *BbHapX* during fungal pathogenic growth. *BbHapX* contributes to fungal survival under the iron-limitation condition. The weakened virulence of the Δ*BbHapX* mutant might partially result from the impaired growth at low iron availability during fungal interaction with the host. Even so, these findings indicate that *BbHapX* functions as a crucial regulator for infection initiation by partially orchestrating FA reserves in conidia.

Many transcription factors are involved in conidial germination (e.g., Forkhead-box protein in Magnaporthe oryzae and C_2_H_2_ zinc-finger protein in Colletotrichum gloeosporioides) (45, 46). Nevertheless, few transcription factors link conidial reserves with their germination capacity. In Aspergillus oryzae, atfA (an ATF/CREB-type transcription factor) controls conidial germination via regulating carbohydrate and amino acid reserves (47). Our study reveals a transcription factor engaged in conidial FA accumulation. Both atfA and HapX belong to the superfamily of bZIP transcription factors. This superfamily mediates comprehensive physiological processes in filamentous fungi. For example, in Podospora anserina, IDI-4 is required for heterokaryon incompatibility (48). In Sordaria macrospora, JLB1 regulates autophagy and is required for vegetative growth and development (49). The yeast AP-1-like transcription factor contributes to the oxidation tolerance of many fungi (e.g., A. nidulans and B. bassiana) (50, 51). Our results update the understanding of the transcriptional control of reserve accumulation in conidia and also extend the roles of bZIP-type transcription factors.

In B. bassiana, BbHapX has a marked transcriptional influence on metabolism during fungal development and acts as a regulator of oleic acid biosynthesis (Fig. 2A). BbHapX regulates two FA desaturases of the OLE pathway which is responsible for oleic acid synthesis. In yeast, activation of the OLE pathway involves transcription factors Mga2 and Spt23 (14). The transcription factor Mga2 is analogous to the sterol regulatory element-binding protein transcription factors in mammalian cells (52). In mammalian cells, FA synthesis is under the control of the basic-helix-loop-helix-leucine zipper family of transcription factors (53). In A. flavus, FarA, a Zn_2_-Cys_6_ transcription factor, positively controls the stearic acid desaturase genes required for the oleic acid biosynthesis (15). In our study, BbHapX acts as a direct transcriptional regulator of the *BbOle1* gene. Desaturase involved in FA metabolism (e.g., Ole1) requires iron as a cofactor for catalytic activity (54). In B. bassiana, exogenous iron has no recovery effects on the impaired growth of the *HapX*-deficient mutant grown on stearic acid. This result reveals that *BbHapX* mediates the desaturation of stearic acid, which is independent of the *HapX*-mediated iron uptake. The present study revealed a new transcription factor controlling oleic acid biosynthesis and suggested that the UFA synthesis pathway is conserved while its transcriptional regulation process varies among organisms.

As revealed, the HapX-Ole1 pathway (HOP) contributes to the maintenance of the plasma membrane integrity of the germinating conidia (Fig. 3D and Fig. 5B). Additionally, the plasma membrane is a critical barrier for conidial survival in nature, and any impairment to the membrane results in the loss of conidial viability (55, 56). For instance, chitosan inhibits conidial germination via disruption of conidial membrane integrity (57). Membrane fluidity is determined by the lipid type, acyl chain composition, and sterol content, which ultimately determine membrane integrity and cell viability (58). In this study, the impaired membrane integrity was restored by exogenous UFAs, particularly by oleic acid. Thus, the HOP controls membrane integrity mainly by maintaining the homeostasis of oleic acid. Similar results have been observed in other microorganisms. For example, Helicobacter pylori, a Gram-negative pathogenic bacterium, relies on UFA biosynthesis for maintaining membrane architecture and function (59). The ratio of saturated to unsaturated acyl chains is an important factor determining membrane fluidity (14). To date, it is well known that UFAs contribute to plasma membrane integrity and determine cell responses to environmental stresses (e.g., hypersaline stress) (60). In budding yeast, *Ole1* expression is significantly upregulated by cadmium stress, and its overexpression confers enhanced cadmium resistance to cells resulting from increased membrane fluidity and integrity (61). In Candida parapsilosis, a yeast pathogen of humans, *Ole1* significantly contributes to fungal virulence through regulating the UFA production for cell membrane biosynthesis (62). Our data provide evidence that UFA homeostasis is required for membrane integrity during conidial germination and fungal infection processes. This finding helps explain why the UFAs supplied by the biosynthesis pathway are critical for the pathobiology of filamentous mycopathogens.

Phospholipids mainly play structural roles in membranes, including phosphatidylcholine (PC), phosphatidylethanolamine (PE), phosphatidylinositol, phosphatidylserine, phosphatidylglycerol, and phosphatidic acid (63). The phospholipid constituents shape the physicochemical traits of the membrane, including fluidity, thickness, rigidity, etc. (64). For example, PC is an important membrane component of vegetative cells and critical for maintaining membrane integrity. In *M. robertsii*, the PC biosynthesis pathway and its products are required for membrane integrity and contribute to fungal development and virulence (65). Triacylglycerol biosynthesis supplies phosphatidic acid and diacylglycerol for phospholipid synthesis and stabilizes the phospholipid compositions of plasma membranes during fungal growth, differentiation, and virulence of *M. robertsii* (66). In B. bassiana, the HOP has a substantial effect on the steady state of membrane phospholipids (e.g., PC and PE) (Fig. 7). Defects in the membrane integrity of the HOP-null mutants are due to the loss of phospholipid homeostasis. It is well known that FAs enter into the triacylglycerol biosynthesis pathway in the form of acyl coenzyme A (acyl-CoA) and then appear as hydrophobic chains in phospholipids (67). Together, these results develop an overview of maintenance of phospholipid homeostasis in filamentous fungi, involving three coupled processes of FA, diacylglycerol, and phospholipid synthesis. BbHapX adopts the HOP as a principal route to balance conidial levels of FAs and phospholipids. However, *BbHapX* has a greater influence on conidial germination, lipid homeostasis, and virulence than *BbOle*. This implies that as a transcription factor, BbHapX might regulate other potential pathways beyond the HOP to maintain membrane homeostasis and conidial physiology.

**FIG 8:**
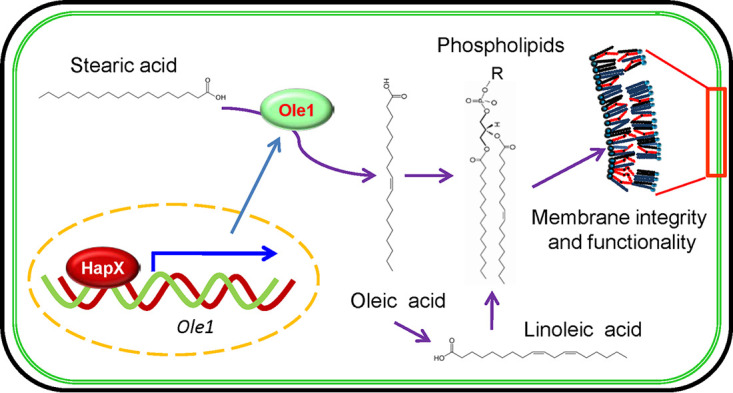
Schematic view of the *HapX*-mediated homeostasis of fatty acid/phospholipids in B. bassiana conidia. HapX functions as a transcription factor of the Δ9-fatty acid desaturase gene (*Ole1*). Ole1 converts stearic acid into oleic acid, which could be further catalyzed into linoleic acid. Both unsaturated fatty acids (UFAs) act as the hydrophobic chains of phospholipids, which are essential for the integrity and functionality of the cytomembrane. The HapX-Ole1 pathway plays an important role in maintaining the UFA flux required for conidial reserves and membrane functionality. R, polar group.

FA/lipid metabolism has been thought to form a coevolutionary network between insect host and entomopathogenic fungi (68). In addition to its conserved roles in fungal adaptation to ambient iron availability, transcription factor HapX is a master determinant for virulence that acts to initiate fungal infection via regulating the UFA biosynthesis pathway to maintain conidial lipid homeostasis and membrane functionality (Fig. 8). The virulence contribution of *HapX* is due to its participation in conidial germination and growth of invasive hyphae. The surface of the insect cuticle is a nutrient-deficient environment. Involvement of endogenous nutrients in infection initiation is an evolutionary adaptation to such selective pressure. This study not only establishes the transcriptional regulation of FA metabolism but also expands our understanding of the diverse mechanisms involved in the initial stage of fungal invasion of hosts.

## MATERIALS AND METHODS

### Strains and culturing conditions.

The WT strain of B. bassiana ARSEF2860 was routinely maintained on Sabouraud dextrose agar at 25°C (22). For plasmid propagation, Escherichia coli DH5α (Invitrogen, Carlsbad, CA, USA) was cultured in Luria-Bertani medium supplemented with an appropriate antibiotic as the selection reagent. During the fungal transformation procedure, Agrobacterium tumefaciens AGL-1 was used as a donor strain and cultured in yeast extract broth medium. Czapek-Dox agar was used as a defined medium in this study. When omitting iron, the medium was named as minimal nutrient agar (MMA).

### Bioinformatics identification of BbHapX and generation of its mutants for functional analysis.

A. nidulans HapX (GenBank no. AB052971) was used to query potential homologs in B. bassiana (37) with a local BLAST program. The homologs in other fungi were downloaded from the NCBI database, and their domains were recognized through the online portal SMART (http://smart.embl-heidelberg.de) (69).

Disruption and complementation of *BbHapX* were accomplished as previously described (22). The gene disruption vector was constructed as follows: the upstream (1.46-kb) and downstream (1.54-kb) sequences of the BbHapX open reading frame (ORF) were amplified by PCR using the primer pairs P_H_1/P_H_2 and P_H_3/P_H_4 (see Table S1 in the supplemental material), respectively. The PCR products were purified and individually ligated into the EcoRI/BamHI and BglII sites of p0380-bar (conferring resistance to ammonium glufosinate), using the ClonExpress II one-step cloning kit (Vazyme Biotech, Nanjing, China). The resultant vector was designated p0380-BbHapX-KO. For gene complementation, the full ORF sequences of *BbHapX*, together with their corresponding promoter regions (amplified with the primer pair P_H_7/P_H_8), were cloned into the vector p0380-sur-gateway (conferring resistance to chlorsulfuron), which was then transformed into the Δ*BbHapX* mutant strain to generate the complemented strain.

10.1128/mSystems.00695-20.4 TABLE S1 Primers used for molecular manipulation in this study. Download Table S1, DOCX file, 0.03 MB. Copyright © 2020 Peng et al. 2020 Peng et al. This content is distributed under the terms of the Creative Commons Attribution 4.0 International license.

All fungal transformation experiments were performed by *Agrobacterium*-mediated methods. The putative mutants were screened by PCR with the primer pair P_H_5/P_H_6 and further confirmed by Southern blot analysis with a DIG DNA labeling and detection kit (Roche, Germany). A fragment (319 bp) amplified with the primer pair P_H_9/P_H_10 (Table S1) was used as a template to prepare the probes.

### Transcriptomic analysis of the BbHapX-mediated gene expression.

A global transcriptomic analysis was performed on the WT and Δ*BbHapX* mutant strains as previously described (20).

Total RNA was extracted from the mycelia cultured on SDAY plates for 3 days at 25°C and constructed into two libraries which were analyzed on the Illumina HiSeq X Ten platform at Vazyme Biotech Co., Ltd. (Nanjing, Jiangsu, China). Each library was repeated twice in independent experiments.

All clean reads were mapped onto the genome database of Bb2860 (37) using the HISAT program (70). All mapped genes were normalized in terms of the expected number of fragments per kilobase of transcript sequence per million base pairs sequenced, using Cufflinks software (71). The Cuffdiff method was used to search the differentially expressed genes (DEGs) between two libraries, using the threshold of the *q*-value of <0.05 (5% false discovery rate) and an absolute value of log_2_ ratio (fold change) of >1 (72). Enrichment analysis was performed on the DEGs using the online FungiFun2 portal (https://elbe.hki-jena.de/fungifun/) (73), using the threshold of the corrected *P* value <0.05.

### FA extraction and analysis.

Conidial free FAs were extracted and analyzed as previously described (10). Briefly, 100 mg conidia (7 days old on SDA plates) was dispersed in 2.5 ml water, and then 5.0 ml chloroform and 2.5 ml methanol were added to suspension. The resulting suspension was kept at −20°C for 2 h. Then, 1 ml chloroform and 2 ml methanol were added to the suspension, followed by mixing. After stratification for 1 h, the bottom layer of the liquid was transferred into a tube and dried on a Termovap sample concentrator. The sample was dissolved in 1 ml *n*-hexane and 0.25 ml sodium methoxide (0.5 M), and the methylation reaction was performed at 55°C for 30 min. The *n*-hexane layer was collected and dried. Finally, the methylated FAs were dissolved in 120 μl *n*-hexane.

Total fatty acid methyl esters (FAME) were analyzed on a Focus series gas chromatograph (Thermo Scientific) coupled to a DSQ2 mass selective detector (Thermo Scientific). Peak areas were recorded using HP ChemStation software (version D.01.02.16, 2004). FAME were identified and quantified using Supelco 37 component FAME mixture as a standard (catalog no. 47885U) (Sigma).

### Lipidomic analysis.

Total FAs and lipids were extracted following the above methods. Samples were separated by ultrahigh-performance liquid chromatography and analyzed on a Q Exactive Plus mass spectrometer (Thermo Scientific). Lipid secondary identification and quantification were performed using the software LipidSearch v.4.1.30 (Thermo Scientific). The mass content of each identified molecule was quantified using a primary ion mass spectrum. The differentially changed molecules between the WT and mutant strains were screened with Student’s *t* test at a threshold of *P* < 0.05. There were three independent replicates for each strain.

### Phenotypic assays and feeding tests with FAs/phospholipids.

Fungal phenotypes were evaluated as described previously (22).

**(i) Conidial germination.** To view conidial germination on the host cuticle, a conidial suspension (10^7^ cells/ml) was sprayed on the locust hindwings. Water agar (WA) plates containing 1.5% agarose were used to mimic oligotrophic conditions. Conidial suspensions (100 μl) were smeared on the WA plates. After 1 day of incubation at 25°C, the germination percentage was examined.

**(ii) Fungal growth on plate and in culture broth.** For aerial growth, aliquots of 1 μl conidial suspension (10^6^ spores/ml) were inoculated on the nutrient plates and incubated at 25°C. Seven days later, the colony diameter was examined. To control the iron level, MMA was used as the iron-depleted conditions. The iron chelator bathophenanthroline disulfonate (BPS) (0.2 mM) and ferrous iron (30 μM) were included in MMA to generate iron starvation and iron-sufficient conditions, respectively. To determine the effects of stearic acid on fungal growth, the final concentration used was 0.3%.

For preparing culture broth, the agar of MMA was omitted, generating MMB broth. The ion levels were controlled as described above. Conidia were inoculated into broth at final concentration of 10^6^ cells per ml and cultured at 25°C with constant shaking (150 rpm). Six days later, mycelia were separated from broth by filtering and dried by the oven-drying method.

**(iii) Conidial pathogenicity.** Two bioassay methods were used on Galleria mellonella larvae. In the cuticle infection assay, the insects were inoculated by immersing the larvae in the conidial suspension (10^7^ conidia/ml) for 15 s. In the intrahemocoel injection assay, 5-μl aliquots of conidial suspension (10^5^ conidia/ml) were injected into the host hemocoel. All bioassay experiments were repeated three times, and 30 to 35 larvae were used in each replicate. The survival data were recorded and plotted as Kaplan-Meier curves. A log-rank test was used to determine statistical difference between the paired curves.

**(iv) Membrane integrity of fungal cells.** SYTOX Green nucleic acid staining was used as previously described (74). The *in vitro* germlings were cultured by spreading 100 μl conidial suspension (10^7^ conidia/ml) on SDA plates for 12 h at 25°C. The *in vivo* blastospores were prepared by injecting 5 μl of conidial suspension (10^7^ conidia/ml) into G. mellonella for 2 days at 25°C. The blastospores were isolated from the hemolymph by centrifuging at 4°C. Cells were stained with 5 mM SYTOX Green for 10 min at 25°C in darkness. The fluorescent signals were observed under a laser scanning confocal microscope, and the percentage of stained cells (PSC) was recorded.

**(v) Feeding test with FAs/lipids.** To determine whether the impaired phenotypes of the *ΔBbHapX* and Δ*BbOle1* mutants resulted from the reduced FA generation, we supplemented FAs in the above phenotypic assays. When examining conidial germination on WA plates, glucose was used as a favored energy source control. Additionally, ferrous ion (30 μM) or BPS plus stearic acid was included in WA plates and used to evaluate conidial germination. Stearic and palmitic acids were each adjusted to a final concentration of 0.3% (vol/vol). The concentration of oleic and linoleic acids was 0.2% and 0.5‱ (vol/vol), respectively. Three phospholipids (lysophosphatidylcholine [LPC], phosphatidylethanolamine [PE], and phosphatidylcholine [PC]) were set as 0.005 mg/ml. SDA plates without additional phospholipids were used as the blank control, and SDA plates with stearic acid were used as an FA control. In insect bioassay, OA was mixed with conidial suspension by vigorous vortexing.

### Functional analyses of Δ9-fatty acid desaturase *BbOle1*.

To probe the potential targets involved in FA desaturation, a significantly repressed Δ9-fatty acid desaturase (locus tag BBA_07664) was deemed a candidate gene and named *BbOle1* due to its high similarity with *Ole1* in budding yeast (GenBank accession no. P21147).

The biological roles of *BbOle1* in B. bassiana were analyzed through the same strategy used on the *BbHapX* gene. All required primers are also listed in Table S1. All phenotypes of interest were examined using the same methods described under “Phenotypic assays and feeding tests with FA/phospholipids.”

### Detecting the requirement of BbHapX for activation of *BbOle1*.

The promoter of *BbOle1* was amplified with the primer pair P_o_11/P_o_12 (Table S1) and fused to the 5′ terminus of the green fluorescent protein gene (*Gfp*). The hybrid fragment was cloned into the XmaI/BamHI site in plasmid pBbTEF-MCS-sur (22). The resulting vector was individually transformed into the WT and Δ*BbHapX* mutant strains. The green fluorescence intensity in mycelia was determined by Image J software (ImageJ 1.52v; National Institutes of Health, USA) and used to calculate the relative expression level of *Gfp* between two strains.

Additionally, electrophoretic gel mobility shift assay (EMSA) was used to detect the direct binding activity of BbHapX to the promoter of *BbOle1*. To increase its solubility, *BbHapX* was fused to the thioredoxin gene. The *BbHapX* coding sequence was amplified from cDNA by PCR using the primer pair P_H_F/P_H_R (Table S1) and cloned into the BamHI and EcoRI sites in pET32a (Novagen) expression vector. The resultant plasmid was transformed into the E. coli Rosetta DE3 strain. The protein was purified through Ni-chelating affinity chromatography and stored at −80°C until use. EMSAs were performed according to previously established methods (75, 76). Briefly, the *BbOle1* promoter for the EMSA was prepared by PCR with the primers P_o_13/P_o_14 (Table S1). The reaction system (20 μl) was established in a 1:10 dilution of binding buffer [poly(dI-dC), 50% glycerol, 1% OP-40,1 M KCl, 100 mM MgCl, 200 mM EDTA (pH 8.0)], promoter (400 ng), and purified BbHapX protein (0 to 2.7 μg). The mixture was incubated for 30 min at 37°C and analyzed on 6% polyacrylamide gels. The nucleic acids were stained with ethidium bromide, and the shifted bands were visualized under a UV lamp.

### Data availability.

The sequence data have been deposited in the NCBI Gene Expression Omnibus (accession no. GSE141409).
