# Supplementary figures and images for: HapX, an Indispensable bZIP Transcription Factor for Iron Acquisition, Regulates Infection Initiation by Orchestrating Conidial Oleic Acid Homeostasis and Cytomembrane Functionality in Mycopathogen Beauveria bassiana
Source: mSystems. 2020 Oct 13;5(5):e00695-20. doi: 10.1128/mSystems.00695-20 (PMC7567583; doi:10.1128/mSystems.00695-20)

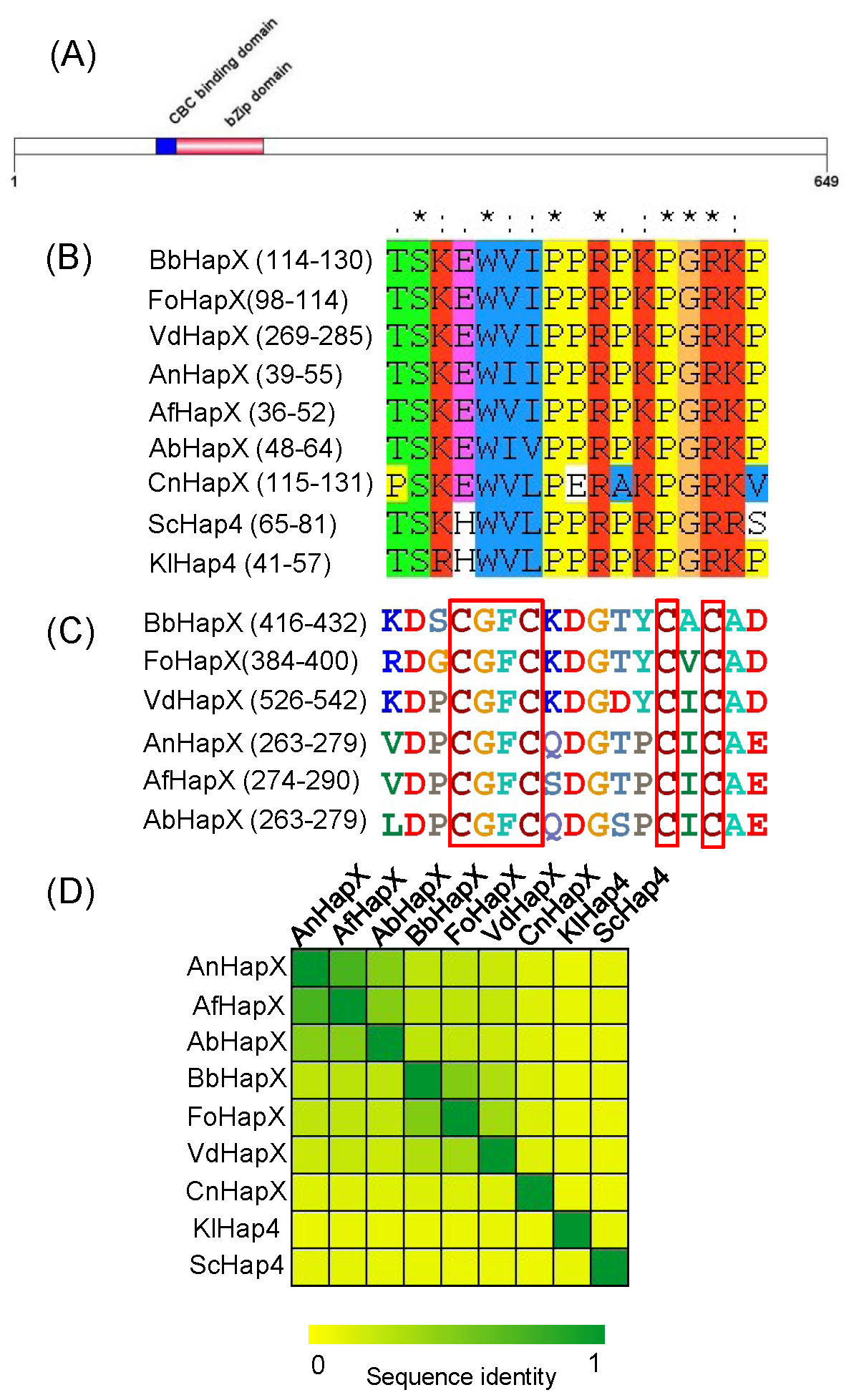

Supplement: FIG S1 [file mSystems.00695-20-sf001.tif]

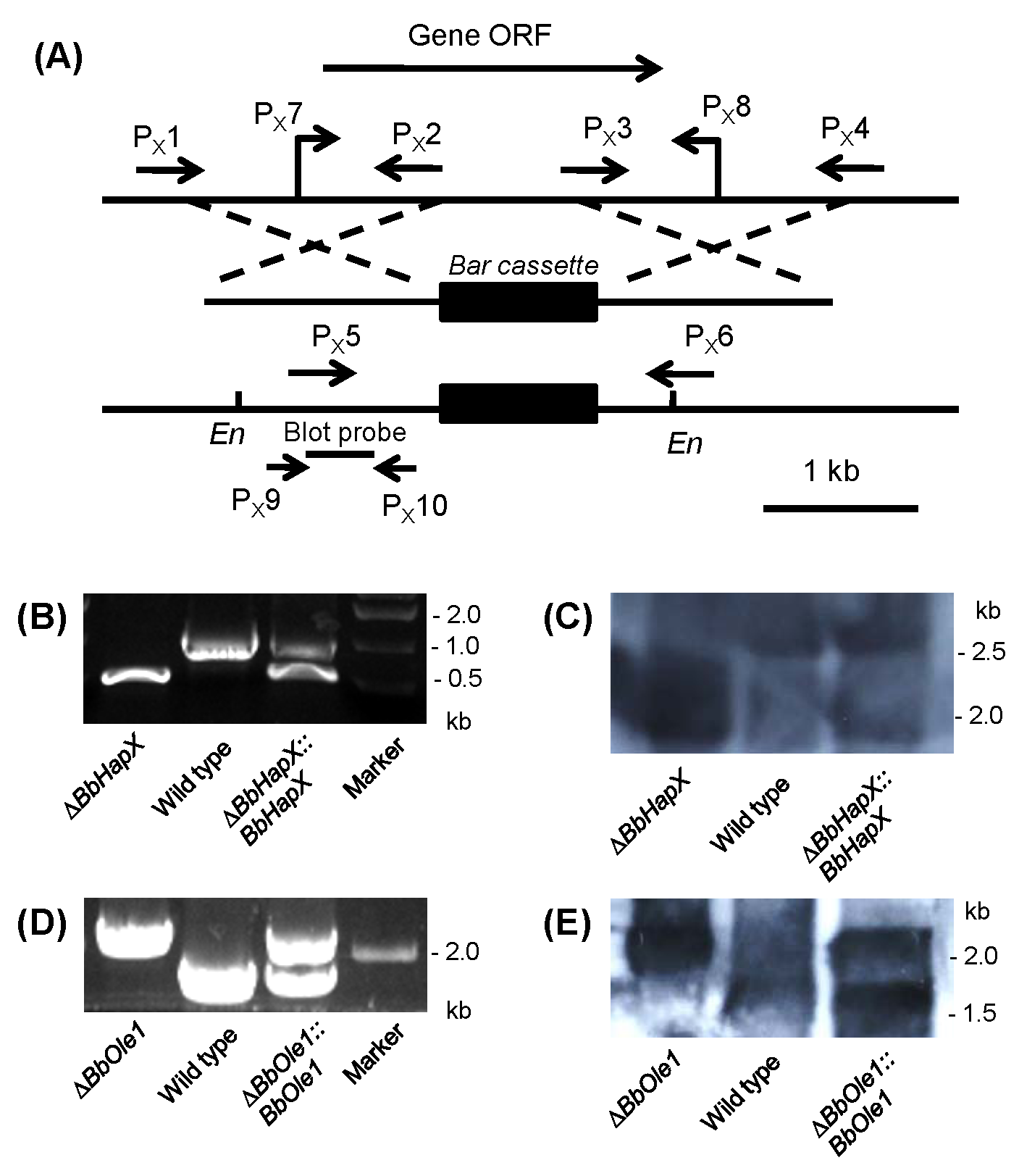

Supplement: FIG S2 [file mSystems.00695-20-sf002.tif]
