# Supplementary material for: HapX, an Indispensable bZIP Transcription Factor for Iron Acquisition, Regulates Infection Initiation by Orchestrating Conidial Oleic Acid Homeostasis and Cytomembrane Functionality in Mycopathogen Beauveria bassiana
Source: mSystems. 2020 Oct 13;5(5):e00695-20. doi: 10.1128/mSystems.00695-20 (PMC7567583; doi:10.1128/mSystems.00695-20)
Supplement: TABLE S1 [file mSystems.00695-20-st001.docx]

**Table S1 Primers used for molecular manipulation in this study.**

| **Primer name** | **Sequence (5′–3′) *^a^*** | **Purpose of use** |
| --- | --- | --- |
| bZip transcription factor (*BbHapX*) | | |
| P_H_1 | TGGGCCCGGCGCGCCGAATTCTCACAAGGATGAGGAAATGC | Obtaining upstream flanking sequence |
| P_H_2 | TGGCTGCAGGTCGACGGATCCGCTGATTTGTGGGGCTACTG |  |
| P_H_3 | GACCCATGGCTCGAGTCTAGACCAATGGAGGGACCCAAAAT | Obtaining downstream flanking sequence |
| P_H_4 | GGTGGTGGTGGCTAGCGTTAACTCTGTCTTGACGACGGGGAT |  |
| P_H_5 | GCCTCTGCTACGACACATAA | Confirming the candidate transformants |
| P_H_6 | CGTCATGGAAATTTTGGGTC |  |
| P_H_7 | ATCCGTCGACCTGCAGCCAAGCTTGCGGAGCGGAGAGTTT | Amplifying *HapX* for complementation |
| P_H_8 | ACACTAGTCAGATCTTCTAGTGTGAATAGACCGAAACCTCCCT |  |
| P_H_9 | ATACAAAACGCCCACCAC | Probe preparing in Sourthern blot |
| P_H_10 | CTACTGGGCTCGGATGTC |  |
| P_E_F | CGGGATCCATGGCCAGCACCGCTGCA | Heterologous expression of *BbHapX* |
| P_E_R | CGGAATTCGCATTCCCGTCCAAAGCGG |  |
| Δ9-fatty acid desaturase (*BbOle1*) | | |
| P_O_1 | TGGGCCCGGCGCGCCGAATTCATAGGTGTCGCGGGCG | Obtaining upstream flanking sequence |
| P_O_2 | TGGCTGCAGGTCGACGGATCCATGGCCAGCCGCTAGC |  |
| P_O_3 | GACCCATGGCTCGAGTCTAGACCAATGGAGGGACCCAAAAT | Preparing downstream flanking sequence |
| P_O_4 | GGTGGTGGTGGCTAGCGTTAACTCTGTCTTGACGACGGGGAT |  |
| P_O_5 | GCCTCTGCTACGACACATAA | Confirming the transformants |
| P_O_6 | CGTCATGGAAATTTTGGGTC |  |
| P_O_7 | ATCCGTCGACCTGCAGCCAAGCTTGCGGAGCGGAGAGTTT | Obtaining the entire gene for complementation |
| P_O_8 | ACACTAGTCAGATCTTCTAGTGTGAATAGACCGAAACCTCCCT |  |
| P_O_9 | ATACAAAACGCCCACCAC | Preparing probe for Southern blot |
| P_O_10 | CTACTGGGCTCGGATGTC |  |
| P_O_11 | ATTGGATGTCAAATGTGTAGGCGGAGCGGAGAGTTT | Amplifying the promoter of *BbOle1* for transcription activity |
| P_O_12 | TCCTCGCCCTTGCTCACCATATGGCCAGCCGCTAGC |  |
| P_O_13 | ATAGGTGTCGCGGGCG | Amplifying the promoter of *BbOle1* for gel shift test |
| P_O_14 | ATGGCCAGCCGCTAGC |  |

*a*: The underlined sequences in primers are required for cloning the PCR products into plasmids by homologous recombination
